# MQTTEEB-D: A Real-World IoT Cybersecurity Dataset for AI-Powered Threat Detection in MQTT Networks

**DOI:** 10.1016/j.dib.2025.111897

**Published:** 2025-07-18

**Authors:** Abderrahmane Aqachtoul, Khaoula Karam, Abderrahmane Elamrani, Mehdi Najib, Najat Rafalia, Mohamed Bakhouya

**Affiliations:** 1 International University of Rabat, College of Engineering and Architecture, LERMA Lab & TICLab, Sala Al Jadida, Morocco; 2 Smart Systems Laboratory (SSLab), National School of Computer Science and Systems Analysis (ENSIAS), Mohammed V University in Rabat, Rabat 10000, Morocco; 3 Ibn Tofail University faculty of sciences in kenitra, IT department, Morocco

**Keywords:** MQTT, IoT security, Intrusion detection system, Cyber threats, Machine learning, Deep learning, Network traffic analysis, Real-time attack execution, Data preprocessing, Post-quantum cryptography, Implementation security

## Abstract

In this paper, we introduce the framework and its experimental design, which was used to elaborate the MQTTEEB-D dataset and to execute real-time MQTT-based attacks while collecting traffic data. The MQTTEEB-D dataset is a practical real-world data set for intrusion detection improvement in MQTT-based IoT networks. Unlike already existing datasets, which are constructed using simulated network traffic, MQTTEEB-D is obtained from a real-time IoT testbed, named MQTTEEB. Various cyberattacks, including Denial of Service (DoS), Slow DoS against Internet of Things Environments (SlowITe), Malformed Data Injection, Brute Force, and MQTT publish flooding, were carried out in real-time while allowing close monitoring of network traffic anomalies. Data was gathered using PyShark and organized into multiple CSV files. To ensure high data quality, we performed pre-processing steps, such as outlier removal, normalization, standardization, and class balance. Several processed forms of data (e.g., raw, cleaned, normalized, standardized), along with detailed metadata are also provided for being used, for instance, to develop and validate AI-driven intrusion detection models.

Specifications Table

**Table:** 

Subject	Computer Sciences
Specific subject area	Cybersecurity, IoT Network Security, Intrusion Detection Systems (IDS), MQTT-based Cyber Threat Analysis, Artificial Intelligence, and Denial of Service Attacks.
Type of data	Raw, Analysed, Filtered, Processed, Cleaned, Metadata JSON files.
Data collection	Real-world dataset was collected from an IoT testbed at EEB laboratory, UIR, Morocco. This testbed, named MQTTEEB, integrated MySignals IoT e-health sensors, Raspberry Pi 4, MQTT brokers, and monitoring systems. Network traffic was captured using PyShark and stored in CSV files. Attacks were performed using a custom-built engine that momentarily requested DoS, SlowITe, Malformed Data Injection, Brute Force, and MQTT Publish Flooding attacks. The data acquired went through preprocessing steps, which includes the elimination of outliers, normalization, standardization, and class balancing.
Data source location	Campus de l'UIR Rabat Salé 11100 - Sala Al Jadida – Morocco.
Data accessibility	Repository name: Mendeley Data • Data identification number: https://doi.org/10.17632/jfttfjn6tr.1 • Direct URL to data: https://data.mendeley.com/datasets/jfttfjn6tr/1
Related research article	ISAAF: IoT Security and Attack Prevention Framework using AI-driven Predictive Analytics, under review, 2025 [14].

## 1 Value of the Data

The dataset will have several added values in promoting further innovation and research in cyber defense strategies for IoT-based communication protocols:

• MQTTEEB-D captures real-time execution of attacks against an IoT-based MQTT environment. Unlike synthetic datasets, it records real-time interactions happening in the network while an attack takes place, hence being valuable for researchers and practitioners.
• MQTTEEB-D includes various forms of cyber threats like DoS, SlowITe, Malformed Data, Brute Force, and MQTT Publish Flood attacks, together with detailed timestamps and accurate labeling. It could be used, for instance, in building, training, and evaluating AI-driven approaches for anomaly detection.
• MQTTEEB-D is well organized and documented allowing seamless reproduction of experiments for benchmarking and comparison purposes.
• MQTTEEB-D was first generated in the form of raw data and then pre-processed, normalized, and standardized, which can be utilized for testing other types of data transformations or feature selections.
• Besides being meant for intrusions detection in MQTT, the MQTTEEB-D could be exploited to train AI-based IDS models for real-world scenarios. These models could be used for real-time and autonomous threats detection and prevention.

## 2 Background

The Message Queue Telemetry Transport (MQTT) protocol has been widely adopted in IoT networks because of its lightweight and efficient publish-subscribe model. However, its built-in lack of security mechanisms leaves it exposed to cyber-attacks, such as DoS, Man-in-the-Middle (MiTM), and unauthorized access attacks [1]. Earlier research identified MQTT security weaknesses, but did not produce a well-structured public dataset. The first public dataset concerning machine learning-based IDS was introduced by MQTTset in 2020, which simulated DoS and unauthorized access attacks [2]. SlowITe focused on investigating direct low-rate DoS attacks targeting MQTT brokers by exploiting session-based vulnerabilities [3]. Building on these works, MQTT-IoT-IDS2020 (2021) extended it with MitM, DoS, and intrusion attempts with improvements in detection of attacks using deep learning [4]. Later, SENMQTT-SET (2022) put forth multi-cascade feature selection, which optimized intrusion detection performance with only the most relevant protocol-level attributes [5]. More recently, the DoS/DDoS-MQTT-IoT dataset [6] was developed, providing real-world DoS and DDoS attack data collected from a physical IoT testbed, emphasizing denial of service scenarios.

While each dataset has contributed extensively to the development of MQTT intrusion detection research, they are based on synthetic simulations and do not encompass some of the inherent complexities of real-world interactions in IoT environments. Again, in most cases, synthetic datasets do not contain attack timestamps, making the traceability of attacks’ impacts on real-time traffic patterns extremely difficult. This inhibits the development of effective IDS solutions based on machine learning.

The MQTTEEB-D dataset was therefore employed to overcome the constraints of existing datasets and to enhance intrusion detection in MQTT-based IoT networks. While most existing datasets are based on simulations, MQTTEEB-D and a few recent real-world datasets, such as DoS/DDoS-MQTT-IoT, capture attacks in real-time together with actual traffic patterns. However, MQTTEEB-D offers greater attack diversity, multi-protocol coverage, and precise timestamping, making it a useful reference for AI-driven intrusion detection research. It contains five attack types, thus making it a good dataset for AI-driven intrusion detection models. In fact, MQTTEEB-D includes precise attack timestamps and actual IoT traffic, providing better ground truth for training IDS. Table 1 presents a comparative summary of the most relevant MQTT attack datasets, illustrating key contributions and limitations.

**Table 1:** Overview of MQTT Attack Datasets.

Dataset	Year	Size (Samples)	Attacks Covered	Environment	Key Features
MQTTset [2]	2020	11,915,716	DoS, Unauthorized Access	Simulated IoT Network	ML-ready dataset, packet capture
SlowITe [3]	2020	∼9,202	Low-rate DoS	Simulated IoT Network	Exploits MQTT protocol weaknesses
MQTT-IoT-IDS2020 [4]	2021	N/A	DoS, MitM, Intrusion	Simulated IoT Network	Bi-flow and Packet-flow features for IDS
SENMQTT-SET [5]	2022	∼34,800,000	DoS, Broker & Subscriber Attacks	Simulated IoT Network	Multi-cascade feature selection
DoS/DDoS-MQTT-IoT [6]	2023	424,716	DoS, DDoS	Real IoT Testbed	Physical deployment, diverse DoS/DDoS patterns
MQTTEEB-D	2025	222813	DoS, SlowITe, Malformed Data, Brute Force, Publish Flood	Real IoT Testbed	Precise attack timestamps, multi-protocol monitoring, live execution

Note: Sample sizes are based on publicly available data or estimates from official publications and repositories. 'N/A' indicates values not explicitly reported.

Furthermore, as cybersecurity threats evolve, the design of intrusion detection systems must account for cryptographic resilience and implementation security, aligning with trends in post-quantum cryptography and secure hardware/software integration [[7], [8], [9]].

## 3 Data Description

MQTTEEB-D was collected from a fully operational IoT testbed, presenting realistic traffic patterns, real-time attack execution, and accurate representation of network behaviors. The dataset is intended to provide support for research work related to AI-driven threat detection, anomaly detection, and cybersecurity research in MQTT-based environments. It was generated from the MQTTEEB test lab, which includes IoT devices, MQTT brokers, a monitoring system, and an engine for the execution of attacks. These latter were injected during normal operations and labeled immediately.

**Fig. 1:**
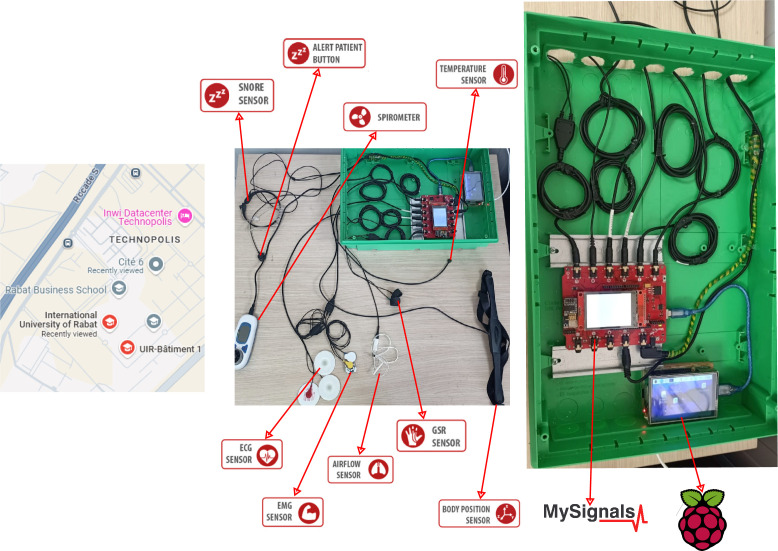
Physical deployment of the MQTTEEB testbed showing the MySignals microcontroller with biometric sensors, the Raspberry Pi 4 (publisher and MQTT broker), and the overall sensor wiring and assembly. Data was transmitted to the ThingsBoard platform (subscriber) hosted on an Ubuntu server (not shown). The setup was deployed at the International University of Rabat (Technopolis).

The MQTTEEB testbed was physically deployed using a real-world IoT setup integrating a MySignals microcontroller connected to multiple biometric sensors and a Raspberry Pi 4 device acting as the publisher and MQTT broker. The system transmitted sensor data to a ThingsBoard platform, installed on an Ubuntu server serving as the subscriber. The testbed enabled the execution of various cyberattacks, including DoS, SlowITe, malformed data, brute force, and publish flood attacks, with precise control over timing and characteristics. This configuration ensured the generation of high-fidelity data for intrusion detection system development. The physical setup is illustrated in Fig. 1.

### 3.1 *Dataset Structure*

The MQTTEEB-D dataset comprises various loops representing attacks in different phases. The data was collected during the four executions of the attack scenarios:

• MQTTEEB-D_dataset_loop_1.csv
• MQTTEEB-D_dataset_loop_2.csv
• MQTTEEB-D_dataset_loop_3.csv
• MQTTEEB-D_dataset_loop_4.csv

The packet-level network traffic dataset was recorded while the respective attack execution was playing out. The main parameters include event timestamps, attack labels, protocol parameters, and several performance metrics. The following dataset was then obtained after merging the above-mentioned individual loops:

• Final dataset: MQTTEEB-D_cleaned_data.csv

This dataset also contains all attack execution cycles to provide information representing the distribution of raw MQTT traffic. This will ensure that attack events are aligned temporally with the corresponding traffic pattern. To have uniform processing of all attack scenarios, Table 2 gives an overview of the dataset logs, addressing basic aspects, such as the start time, the time spent in each loop, and the number of collected samples.

**Table 2:** Dataset Log Information of MQTTEEB-D.

No	Name	Description	Protocol Layer	Data Type
1	timestamp	The time when the packet was captured	TCP	object
2	tcp_flags	TCP flags indicating the status of the packet	TCP	int64
3	tcp_time_delta	The time difference between two successive TCP packets	TCP	object
4	tcp_len	The length of the TCP segment	TCP	int64
5	mqtt_conack_flags	MQTT connection acknowledgment flags	MQTT	object
6	mqtt_conflag_cleansess	Clean session flag in MQTT connection setup	MQTT	int64
7	mqtt_conflags	MQTT control flags, indicating the type of MQTT message	MQTT	object
8	mqtt_dupflag	MQTT duplicate flag, indicating a re-transmitted message	MQTT	int64
9	mqtt_hdrflags	MQTT header flags, used for control purposes	MQTT	int64
10	mqtt_kalive	MQTT Keep Alive value, indicating the interval between client pings	MQTT	int64
11	mqtt_msg	The actual MQTT message content	MQTT	object
12	mqtt_qos	MQTT Quality of Service level	MQTT	int64
13	target	The target label indicating whether the traffic is normal or under attack	Classification	float64

### 3.2 *Description of Data Fields*

The dataset is layered, consisting of TCP-level properties and MQTT flags and control messages, with an extra layer representing either normal or attack traffic. Such a multilayered approach is representative in capturing the network behaviors and the variations associated with the protocol. For instance, network traffic and TCP-level properties, like timestamp, tcp_flags, and tcp_len, assist in offering much more in-depth statistics on packet levels. The field tcp_time_delta measures the delta time between packets; its presence assists in identifying delays or if an attack congested the network. With respect to MQTT protocol-level flags-mqtt_conack_flags, mqtt_conflag_cleansess, and mqtt_qos, serve as key indicators for assessing the stability and reliability of the connection. In addition, mqtt_dupflag and mqtt_hdrflags would show duplicated messages and degradation in headers, which ensures that some prior condition is met regarding malformed data attacks and flooding. Table 3 provides a thorough overview of the most important attributes in the MQTTEEB-D dataset, showcasing their involvement in the TCP and MQTT communication layers.

**Table 3:** Key Features in MQTTEEB-D Dataset.

Log	Start Time	End Time	Duration (HH:MM:SS)	Num. of Samples
MQTTEEB-D_dataset_loop_1.csv	2024-09-09 03:17:53.8393	2024-09-09 11:43:39.8545	08:25:46.0159	137319
MQTTEEB-D_dataset_loop_2.csv	2024-09-09 11:43:39.8766	2024-09-09 14:19:43.2608	02:36:03.3841	42470
MQTTEEB-D_dataset_loop_3.csv	2024-09-09 16:40:13.7705	2024-09-09 18:06:17.1444	01:26:03.3738	21501
MQTTEEB-D_dataset_loop_4.csv	2024-09-09 21:29:57.0083	2024-09-09 23:34:12.4298	02:04:15.4216	21523
MQTTEEB-D_cleaned_data .csv	2024-09-09 03:17:53.8393	2024-09-09 23:34:12.4298	20:16:18.5905	222813

### 3.3 *Exploratory Data Analysis (EDA) Framework*

**Fig. 2:**
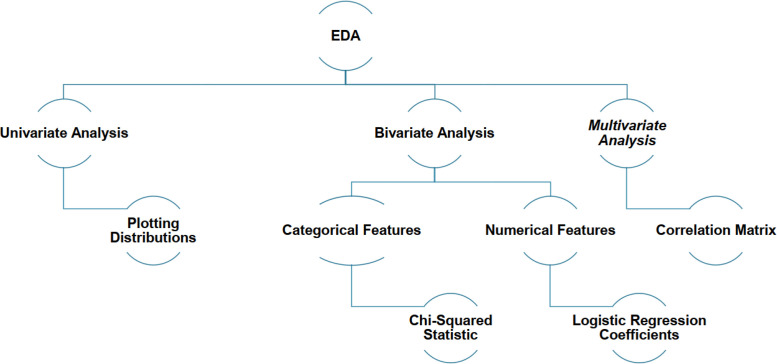
MQTTEEB-D EDA Framework.

The Exploratory Data Analysis (EDA) of the MQTTEEB-D dataset was undertaken to provide an overview and understanding of the dataset. It constitutes an important stage to detect the underlying patterns, feature distributions, and anomalies that may negatively impact the performance of AI-driven attacks detection. In Fig. 2, the three analyses that constitute the EDA framework are shown: univariate analysis, bivariate analysis, and multivariate analysis. Univariate analysis is concerned with knowledge of the distributions of independent variables, which are present in the dataset. This may include graphical displays of variances for such numerical attributes as the length of packets, variation in timestamps, and the control flags of MQTT, which allow the identification of outliers, skewness of distributions, and irregularities in packet transmission behavior.

The relationship of two variables in bivariate analysis helps in detecting dependencies or correlation. Such typically categorical features, like those of the MQTT-specific flags, were analyzed by Chi-Squared statistics aiming to show separation between normal and attack traffic. Numerical features, like time-varying message exchange, were assessed by regression methods to identify traffic variations. Finally, the multivariate analysis involves analyzing multiple aspects of the dataset simultaneously. A correlation matrix was developed. It shows the dependence relationship between the attributes of TCP and MQTT and indicates possible redundancy and strong relationships between attributes. Besides, logistic regression coefficients were found to determine the effect of several variables impacting attack detection, enhancing the interpretability of important network features.

**Fig. 3:**
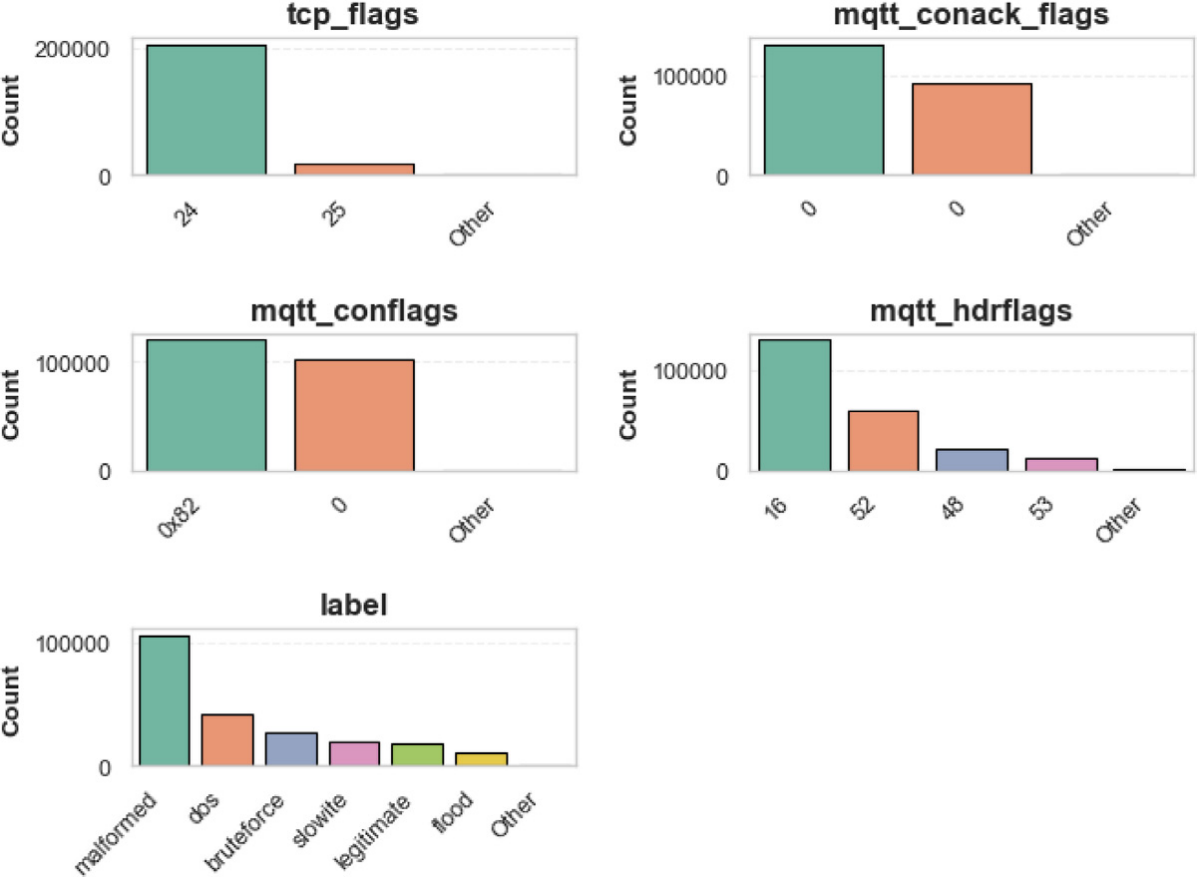
Categorical distribution of individual features.

**Fig. 4:**
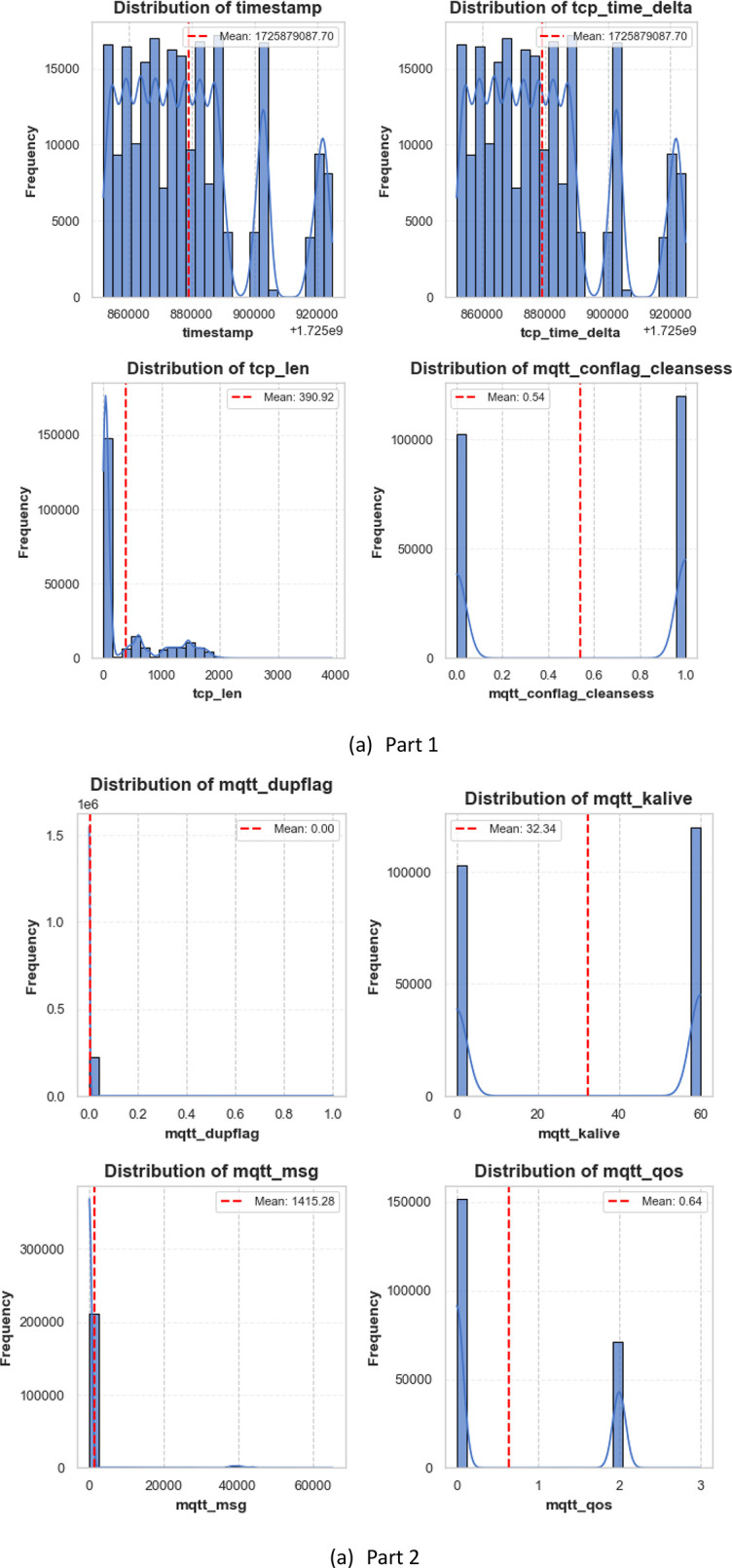
Distribution of individual *numerical* features.

The structured EDA approach ensures a thorough understanding of the dataset structure while shedding insight before conducting feature engineering and models’ training. In fact, the EDA process provided insights into both the structure and specifications of the MQTTEEB-D dataset. This feature exhibits patterns for both numerical and categorical features. The notable findings of the analysis are summarized as follows. Being aware of the distribution of each feature affirms that the detection of outliers, the characterizations of sampling imbalances, and dominating features turn out to be simpler. The distributions of numerical and categorical variables are depicted in Figs. 3,4.

• Characteristics affecting numerical data:
  ○ tcp_len possesses a long-tail distribution; meaning most of the packets sent are small-sized; a few outliers could show very high traffic.
  ○ mqtt_msg has the same distribution whereby the size variability of MQTT messages is very large.
  ○ mqtt_kalive and mqtt_qos show one peak each, putting across a point that the communication in MQTT is done with only a few predefined configurations.
• Characteristics pertinent to categorical data:
  ○ For the tcp_flags, the most frequent flag is 0×0018, which mainly refers to the acknowledgment usually handled in typical TCP, whereas under attack situations, other flags emerge.
  ○ For the mqtt_conflags and mqtt_hdrflags, many values are clustered near other points suggesting a behavior that follows the real-world protocol and can distinguish between attack types.

**Fig. 5:**
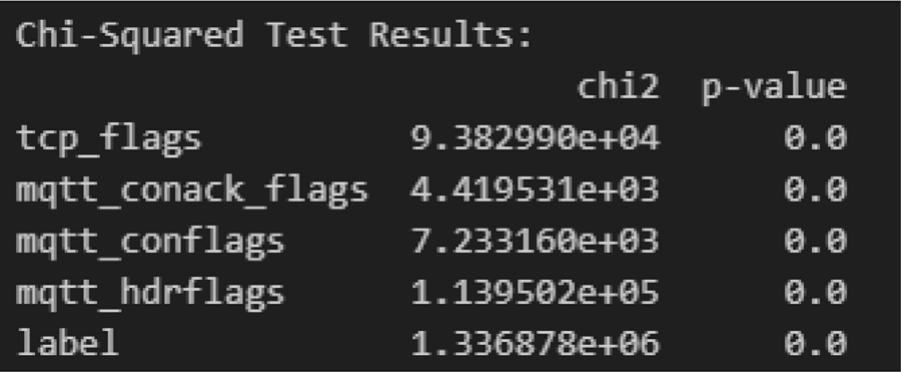
Chi-Squared Results.

Statistical tests and categories associations were made to assess individual feature contributions toward threats detection. As illustrated in Fig. 5, with great statistics, Chi-Squared Test showed that the MQTT control flags contribute enormously toward attack detection, with the most salient features recorded as follows:

• mqtt_hdrflags (χ² = 113,950.20): very high positive correlation concerning attack labels, thus allowing its capability of being discriminative features.
• tcp_flags (χ² = 93,829.90): of intense importance in distinguishing TCP traffic from HTTP normal traffic.
• mqtt_conack_flags and mqtt_conflags: analysis and statistically significant, thus validating their utility in attack-based classification.

**Fig. 6:**
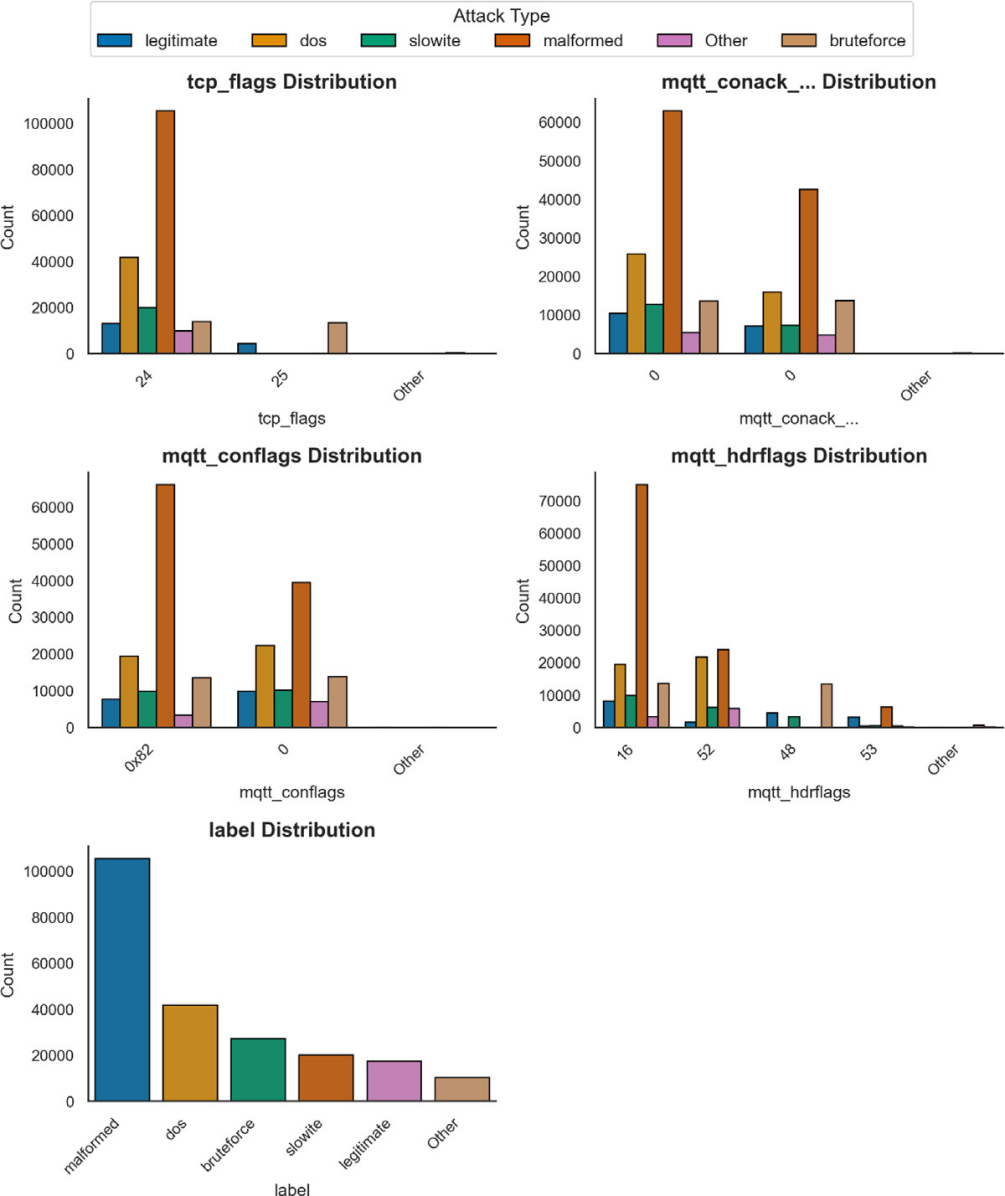
categorical features distribution.

Besides that, Fig. 6 exemplifies the distribution of categorical features across attack labels. Specific MQTT control and acknowledged flags appearing in malicious traffic occurred more frequently, which also attests to their applicability in cybersecurity.

The EDA results show that the characteristics of MQTT itself dictate the differential approaches in detecting an attack. This support is favored for their application in AI-driven approaches for IDS. However, positive preprocessing, balancing, and selection processes should also be put in place before the deployment of AI models.

The next section discusses the procedures followed, starting by handling imbalance, followed by transformations, and finishing with training optimization.

### 3.4 *Data Preprocessing*

**Fig. 7:**
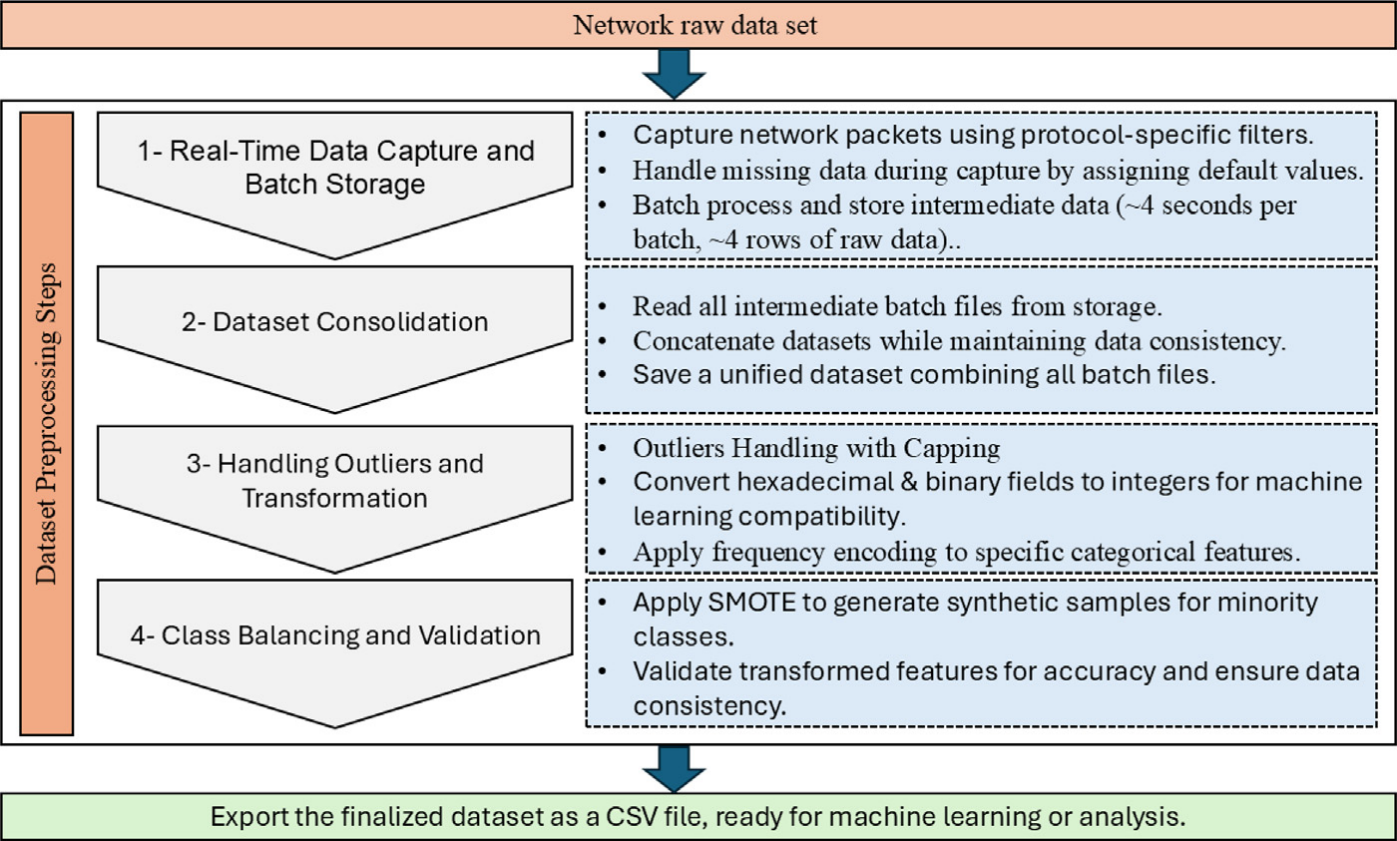
Dataset Preprocessing Steps.

Fig. 7 breaks down complete processing into four phases or processes, containing real-time data capture and batch storage, dataset convergence, outlier handling and treatment, and class balancing and validation. Each of these processes is helpful in cleaning the dataset without losing any important information. The first phase of this process is the actual capture of network packets, which are filtered through a specific operational protocol, with entry placeholders assigned to missing data. This step stores data in batch-file format, ready for further processing. The second phase concerns the compilation of the captured data, in which the order of the previously batch-stored data is reformulated into a single continuous clean dataset.

**Fig. 8:**
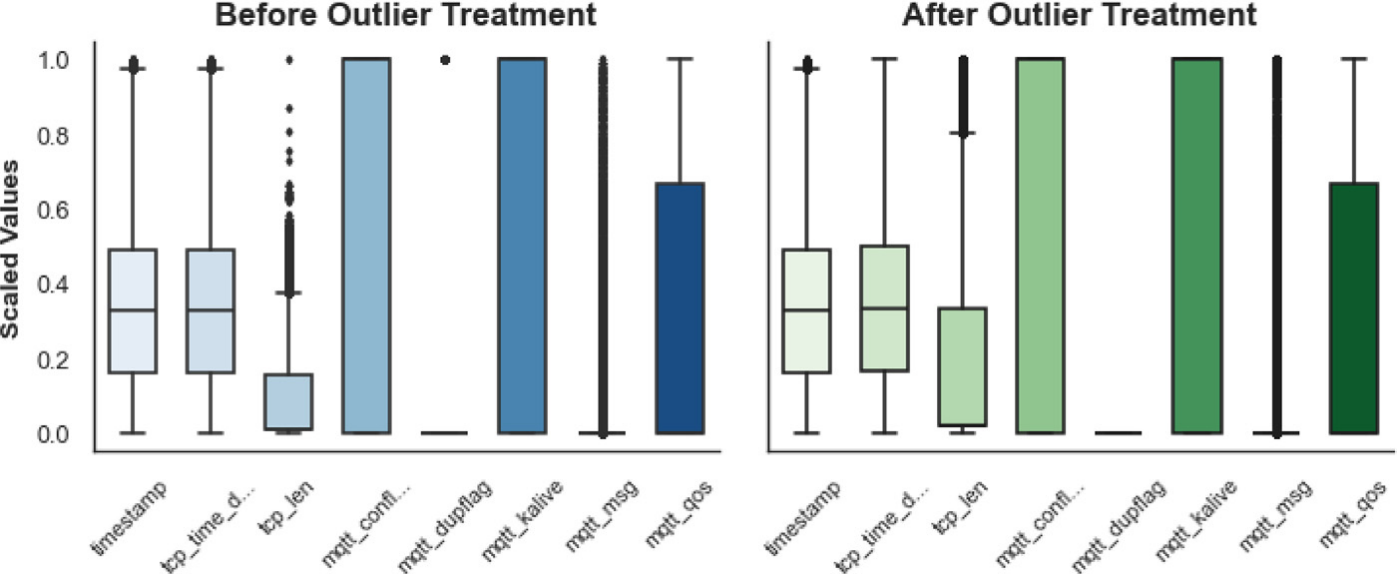
Boxplot of Scaled Numerical Features Before and After Outlier Handling.

**Fig. 9:**
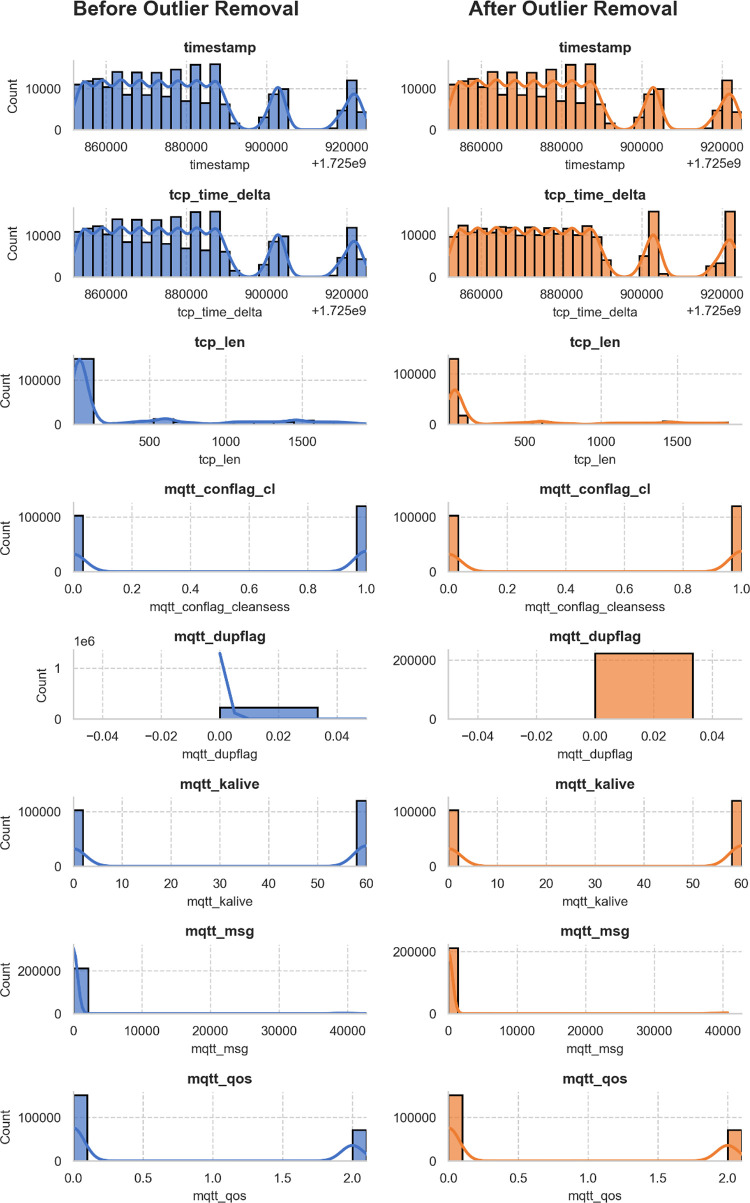
Feature Distributions Before and After Outlier Removal.

The datasets then undergo the subsequent step in the preprocessing pipeline, being outlier handling and processing. The preliminary investigations show the existence of anomalously extreme outliers in tcp_len and mqtt_msg, which will influence models’ performance. Fig. 8 visualizes the distribution of numerical features prior to outlier handling; here, most extreme values outrun far beyond some fine occurrences. The same method of detecting outliers was utilized, employing capping values outside given thresholds, allowing for variability retention. Post-outlier cap processing, as depicted in Fig. 8, displays a noticeable reduction in extreme values, while still preserving comparable distributional characteristics, as seen in Fig. 9.

**Fig. 10:**
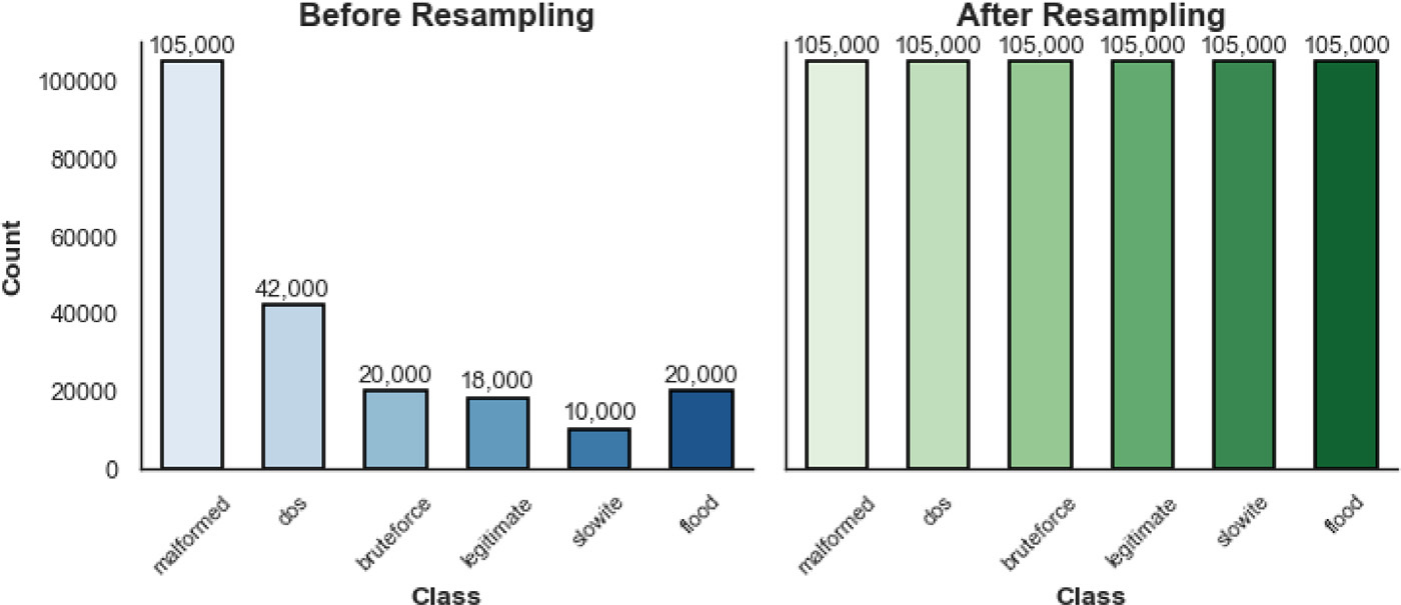
Class Distribution Before and After Resampling.

The bouncing class distributions were another noticed imbalance. Fig. 10 shows the distribution before resampling taking place. The SMOTE oversampling algorithm was applied to synthesize more samples for classes that exhibited a weakness in numbers, thus leading to the generation of balanced datasets. The updated class distribution is shown in Fig. 10, where all classes are fairly and evenly represented.

For sake of compatibility, such fields as tcp_flags, mqtt_conflags, and mqtt_hdrflags have their hexadecimal values converted to integers. The remaining were the binary features, mqtt_conflag_cleansess and mqtt_dupflag, unchanged in form as categorical labels got encoded into numerical variables. Introspective transformations were documented in categorical_processing_metadata.json and label_encoding_metadata.json, which could be used to trace back the performed operations.

Numerical features were further processed by scaling to ensure effective training of AI models. Features with a fixed range, such as mqtt_qos and tcp_flags, were scaled using min-max scaling from 0 to 1, whereas those that had proven upper-correlation variance, such as tcp_len and mqtt_kalive, were standardized using z-score transformation. Such methods seem to be geared toward enhancing models’ performance by ensuring a more comparable distribution of features.

**Fig. 11:**
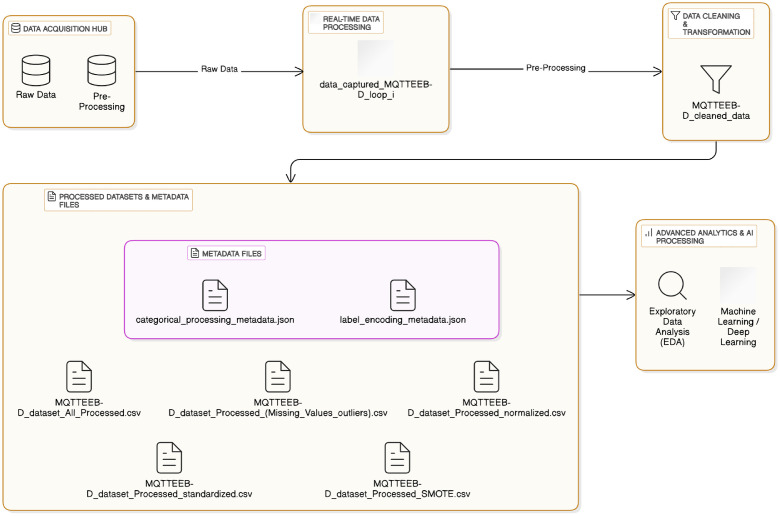
Comprehensive End-to-End Data Processing & AI Pipeline for Cybersecurity Applications.

After pre-processing, the generation of multiple dataset versions was something to consider in support of future AI-driven workflows. The processed datasets and metadata files play various roles in training AI-driven models and for data interpretation as well. The last fully cleaned and manageable version of the processed dataset (MQTTEEB-D_dataset_All_Processed.csv) is now cleanly processed and ready for immediate feeding into cybersecurity applications. Each version of the dataset meets different preprocessing needs (see the Fig. 11):

• MQTTEEB-D_dataset_Processed_(Missing_Values_outliers).csv: missing value imputation and outlier-handled data.
• MQTTEEB-D_dataset_Processed_normalized.csv: feature scaled using Min-Max normalization, perfect for algorithms that need normalized input.
• MQTTEEB-D_dataset_Processed_standardized.csv: transactional variables standardized using Z-score; good for algorithms that need to assume a normal distribution of data.
• MQTTEEB-D_dataset_Processed_SMOTE.csv: The balanced dataset version, which was prepared by means of SMOTE in order to address class imbalance.

## 4 Experimental Design, Materials and Methods

This section highlights a thorough configuration of hardware and software, including IoT devices, attacks generation engine, supervising and data-collection devices. Two custom algorithms were also developed to orchestrate the coordination between real-time execution of attacks, the simultaneous traffic capture, and labeling of attack traces. Accordingly, the developed algorithms justify the proper execution of attacks together with automated data capture and harmonized integration of datasets. We detail attack scenarios and configuration parameters, so the setup can be re-built and deployed in other IoT environments, for testing and evaluation purposes.

### 4.1 *IoT-Based Security Framework for Real-Time Attack Injection & Monitoring*

**Fig. 12:**
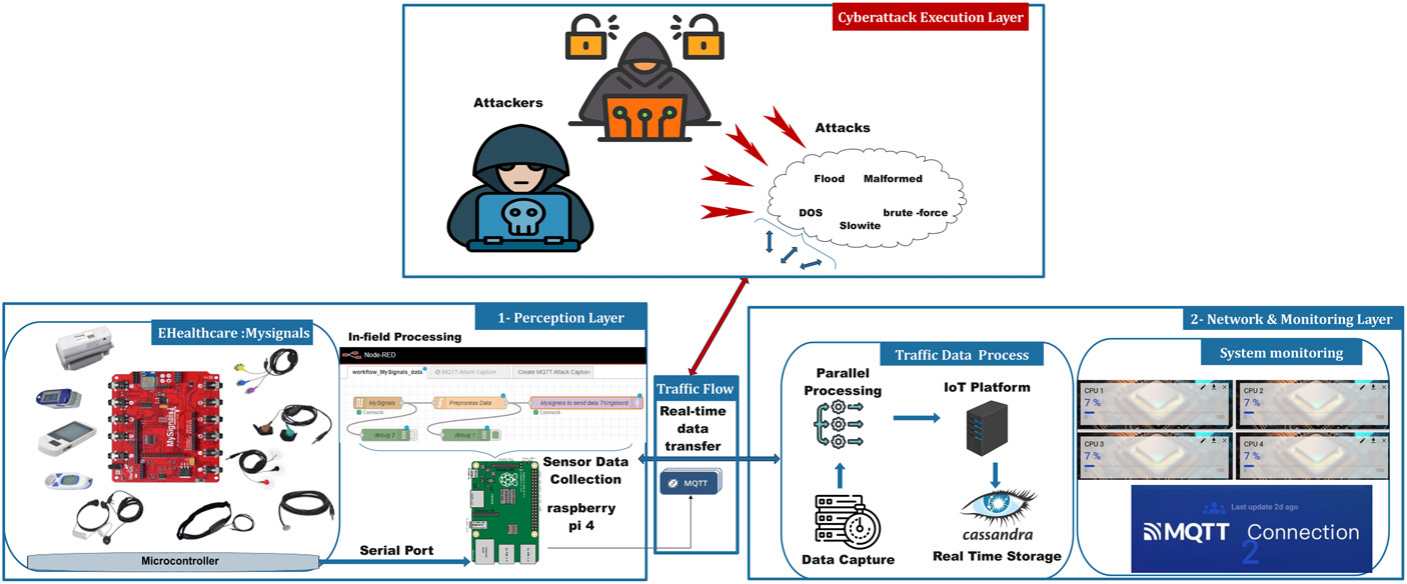
IoT Security Framework for Attack Execution and Monitoring.

To assess attacks that might affect IoT networks, we set up a framework for attack execution and monitoring, capable of injecting and mimicking real-time attacks in IoT-based systems. The proposed framework, as depicted in Fig. 12, involves multi-layered protocols that execute attacks, capture network traffic, and analyze the system behavior accordingly. The perception layer includes IoT sensors and MySignals devices, which are used for real traffic generation. The cyberattack execution layer hosts a built-in attack engine, specifically targeting the MQTT communication protocol with multiple cyberthreats. The network and monitoring layer captures traffic in real time, which uses parallel processing techniques to further process and store labeled traces.

In the perception layer, MySignals e-health sensors are interfaced with a Raspberry Pi 4 to provide continuous data collection; this includes ECG, pulse oximeter, and many other biometric signals, which are processed using Node-RED and transmitted over MQTT to a central monitoring system, developed using Thingsboard IoT platform. The injection engine systematically controls various MQTT-based threats, for instance, a DoS attack overwhelms the network with messages, while a SlowITe attack introduces delays, disrupting legitimate communication. Additionally, it injects malformed data by sending improperly formatted MQTT payloads and uses brute force attacks to breach the broker’s authentication. Each attack is dynamically configured following the CBR (Constant Bit Rate) model with key parameters, such as the number of attacking clients, message size, and transmission rates. In this process, traffic that's been captured undergoes systematic processing by the monitoring and data processing module. Packet analysis is done by PyShark for packet sniffing along with effective filtering and labeling mechanisms. All captured traffic data is stored across multiple CSV files, enabling long-term analysis, retrospective security assessments, and other analytical operations as well.

A feedback loop that correlates attack activity with network behavior ensures synchronization between attack execution and monitoring. Real-time synchronization facilitates the embedding of precise detection and classification within intrusion detection analysis for online attacks. This modularity enhances scalability and reproducibility of experiments, enabling a systematic approach to testing and validating intrusion detection mechanisms.

### 4.2 *Proposed Workflow for MQTT Attack Execution & Data Capture*

**Fig. 13:**
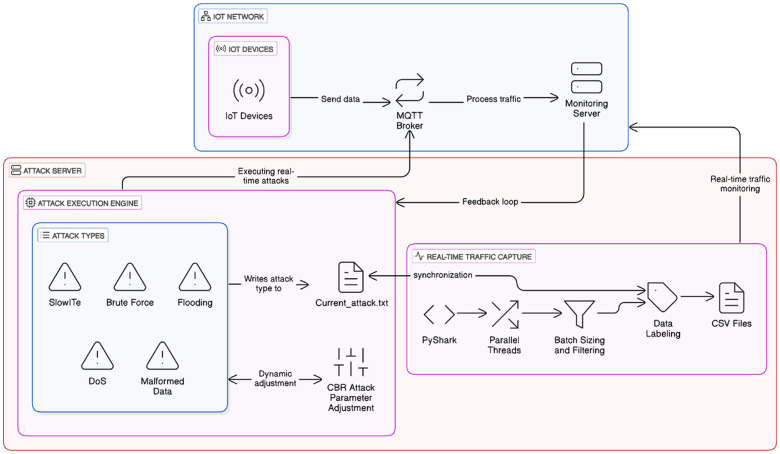
Automated Attack Execution and Real-Time Data Capture workflow.

This section outlines the workflow for automated attack encapsulation and data capture, along with real-time cyber threat evaluation. As shown in Fig. 13, the upper layer of the proposed workflow consists of IoT devices, an MQTT broker, and monitoring servers for traffic processing, while the lower layer is dedicated to executing attacks and capturing and processing data in real time. This layer has its core modules, the attacker execution module and the traffic capture module. The first dynamically performs various MQTT-based attacks, customizing their intensity and running configurations through a CBR-based technique. The attacks include DoS, SlowITe, Brute-Force, MQTT Publish Flood and Malformed Data Injection. Every time an attack is launched, its corresponding metadata is written to current_attack.txt, allowing the monitoring program to match captured packets with the active attack. The traffic capture module, in turn, captures packets with PyShark, through a multi-threaded parallel-processing pipeline. The captured packets are then collected in batch fashion and filtered according to their respective attack types, as recorded in the metadata file. This automated workflow maintains data integrity and supports precise traffic analysis.

Another key feature of this workflow is the feedback loop mechanism, which continuously refines attack execution by leveraging real-time network observations to guide the refinement process. This adaptive control will optimize attacks’ parameters to reflect changes in the condition of the network resulting in more precise experimental evaluations. In fact, executing attacks alongside simultaneous traffic monitoring is essential for evaluating MQTT-based cyber threats. In addition, its modular and scalable architecture allows for the integration of other types of attacks and monitoring extensions, with future developments expected to enhance IoT security research.

**Fig. 14:**
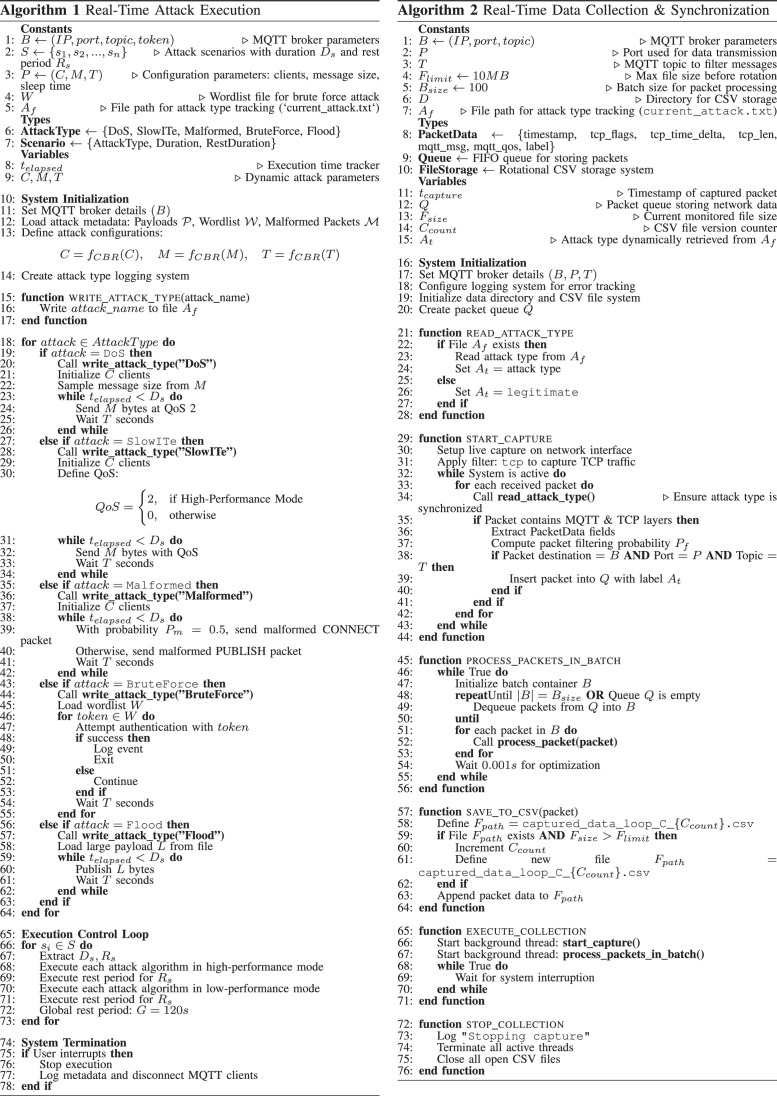
Algorithmic Design for Real-Time MQTT Attack Execution and Traffic Labeling in IoT Networks.

Two algorithms have been developed for automated and adaptive attack execution (see Fig. 14), ensuring events are correctly aligned with the captured network traces. The first enables the launch and control of MQTT-based attacks by following predefined attack scenarios, while dynamically adjusting the execution parameters through a CBR-based mechanism. Each attack follows a predefined sequence and is executed in a controlled environment with varying intensities. The algorithm writes attack metadata to the current_attack.txt file before launching the attack. This ensures that the traffic capture module (Algorithm 2) will correctly label incoming packets. The attack is executed in phases, alternating between high and low-performance attacks with intermittent pauses, simulating real-world attack scenarios. The main functions of the attack execution algorithm are: i) initialization of configurable parameters for the MQTT attacks (number of clients, message sizes, transmitting rates), ii) open attacks with dynamically altered configurations, iii) real-time logging of attack metadata, iv) maintain intermittent rest periods to mimic non-attack traffic conditions, and v) provision of feedback for adjustment of attack strategies according to network conditions being observed.

The traffic capture and synchronization algorithm manage real-time network traffic monitoring during an attack, ensuring accurate labeling of different traffic types by continuously reading an attack metadata file to associate captured packets with their respective attack types. This algorithm utilizes the PyShark packet sniffer and a multi-threaded processing pipeline. Incoming packets are filtered, converted, and labeled according to their respective attack scenarios. To enhance efficiency, the algorithm employs batch processing and parallel execution. Its main functions are: i) real-time packet capture using PyShark, ii) multi-threaded processing to manage high throughput data, iii) batch filtering and attack labeling based on attack metadata, and iv) structuring and storing data for further processing. It is worth noting that the adaptive attack execution module, tightly synchronized with traffic analysis, ensures a high level of accuracy in experimental evaluations, making this approach highly effective for IoT security research.

**Fig. 15:**
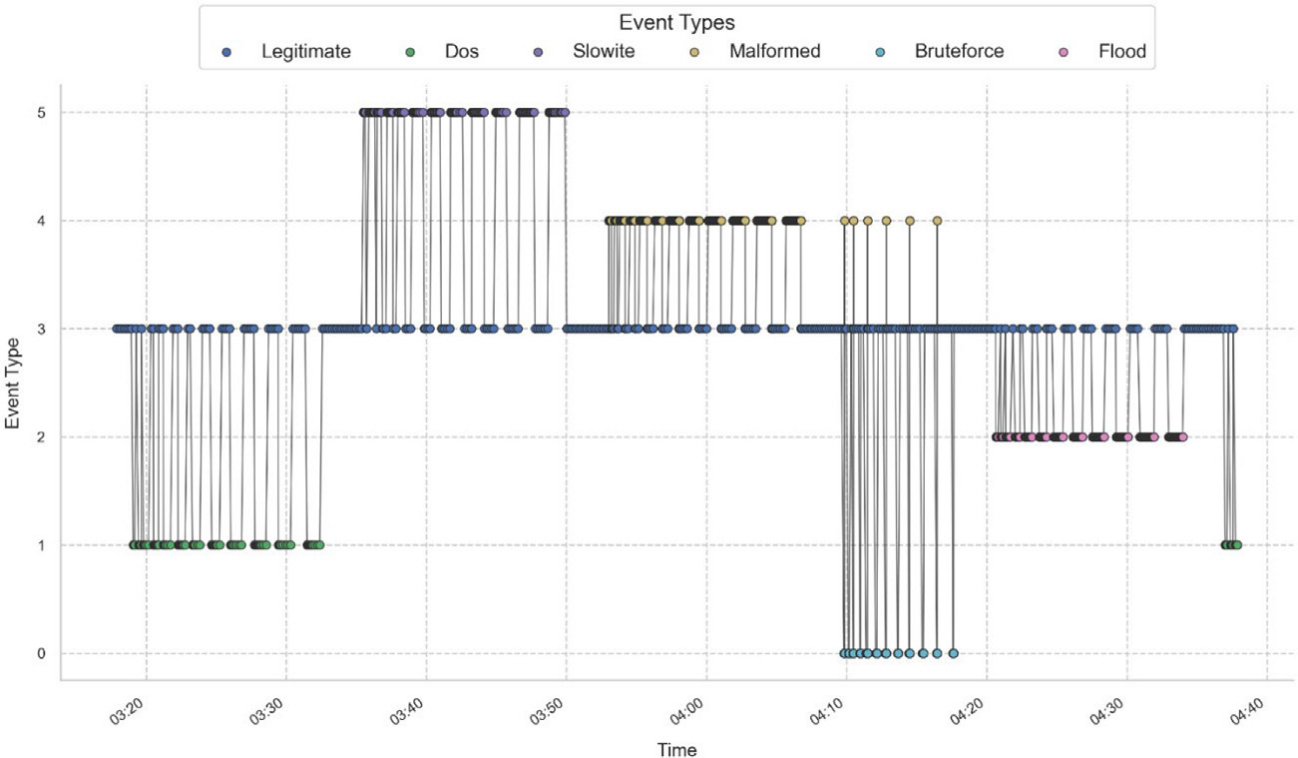
Structured Execution of MQTT Cyberattacks Over Time.

All our experiments follow a general structure with a predefined attack scenario. Each scenario represents a distinct type of cyberattack within the MQTT environment, defined by key parameters such as the number of clients, message sizes, and sleep times, all controlled through a CBR-based mechanism. Table 5 presents attack execution parameters, while Table 6 provides scenario timing and structured execution. An illustration of real-time attack scenario visualization is given in Fig. 15. For instance, Table 5 describes parameters for different kinds of attacks. Each attack is structured and dynamically configurable. It mentions the number of attacks as well as the size distribution of messages, the time between attacks, and the duration of execution. These parameters can be changed dynamically following the CBR mechanism. The first attack scenario is a DoS attack, flooding the MQTT broker with an overload of connections and receiving message floods [10]. The number of attacking clients is dynamically altered from 200 to 100, such that both high-performance and low-performance attack modes could be combined. The initial message size will range from 8000 to 12000 bytes and then be reduced to between 700 and 2000 bytes to simulate varying intensity flooding patterns. Additionally, the sleep time (the interval between sending messages) is dynamically adjusted, ranging from 0.0005s in high-intensity attack mode to 0.01s in low-intensity mode.

**Table 5:** Attack Execution Details for All Scenarios

Scenario	Attack Type	CBR-Adjusted Parameters	Duration (s)	Metadata Used
Scenario 1	DoS	Clients: 200 → 100, Msg Size: 8000-12000 → 700-2000, Sleep Time: 0.0005 → 0.01	41802	No external files
Scenario 2	SlowITe	Clients: 100 → 50, Msg Size: 35000-50000 → 25669-30000, Sleep Time: 0.05 → 0.5	20090	No external files
Scenario 3	Malformed Data	Clients : 200 → 100, Sleep Time : 0.01 → 0.1	105528	Predefined malformed packets
Scenario 4	Brute Force Attack	Clients : 200 → 100, Sleep Time : 0.0005 → 0.01	27377	Uses ‘rockyou.txt’ and ‘usernames.txt’
Scenario 5	MQTT Publish Flood	Clients: 1, Msg Size: 50000, Sleep Time: 0.00001 → 0.001	10213	Uses ‘big.txt’ for large payloads

**Table 6:** Scenarios Time Execution

Scenario	Attack Duration (s)	Rest Duration (s)	Scenario Rest (s)	Total Scenario Time (s)
*Scenario 1*	*10*	*10*	*	*20*
*Scenario 2*	*20*	*20*	*	*40*
*Scenario 3*	*30*	*30*	*	*60*
*Scenario 4*	*40*	*40*	*	*80*
*Scenario 5*	*50*	*50*	*	*100*
*Scenario 6*	*60*	*60*	*	*120*
*Scenario 7*	*	*	*120*	*120*

In contrast, the SlowITe attack scenario is a low-bandwidth, high-impact attack, differing from the typical high-bandwidth, low-impact DoS attack. Unlike a generic DoS attack that overwhelms the target with malicious traffic, SlowITe establishes numerous MQTT connections while transmitting minimal data to each [[11], [12], [13], [14]]. The number of active clients is maintained at 100 during the high-performance attack mode, while in the lower-performance attack mode, the active client count is dynamically reduced, starting at 50. After the initial large message size, typically ranging from 35,000 to 50,000 bytes, it is adjusted to a smaller range of 25,669 to 30,000 bytes to systematically exhaust the broker's resources. The attack progresses gradually, relying on an adaptive sleeping scheme with delays of 0.05s, resulting in slower connection exhaustion that reduces the likelihood of immediate detection.

The Malformed Data attack involves injecting corrupted MQTT packets to exploit vulnerabilities within the protocol [15]. The attack specifically involves testing predefined malformed packets, sending them to generate expected payloads designed to trigger erratic behavior in the broker. Sleep time remains relatively stable, especially compared to flooding attacks, allowing the exploitation of protocol parsing mechanisms to take precedence over exhausting system resources.

The Brute Force Attack operates on the principle that attackers can gain access to a victim's account by systematically testing every possible combination of credentials [16]. The execution framework uses rockyou.txt and usernames.txt datasets, which are relatively popular in real-world brute-force scenarios. This attack incorporates an adaptive sleep time strategy, allowing for both rapid and stealthy execution, with timing ranging from 0.0005s to 0.01s.

Finally, the MQTT Publish Flood attack aims to create a load on the broker processing resources by transmitting huge payloads [17]. Unlike DoS attacks, which rely on multi-client flooding, this attack utilizes a single client, where a lone attacker sends messages to overwhelm the broker's buffer mechanisms, which create heavy congestion. The size of the message is set to be 50,000 bytes, while sleep time varies between 0.00001s to 0.001s, ensuring that both instantaneous burst floods as well as prolonged transmission-saturation scenarios can be covered. This adaptive attack structure allows for systematic scenario adjustments of the several attack scenarios, thereby enabling the generation of a balanced dataset that reflects appropriately real-world cyber threats.

The timeframe(s) for executing attacks are shown in Table 6, providing a structured progression of attack deployments and traffic presentation. Unlike uncontrolled attacks, this structured approach simulates real-world adversarial behavior and thus captures the dynamic interaction between malicious activity and normal network conditions. Crucial in this attack execution framework are the gradual escalation of the attack duration and the rest of the durations, permitting a layered representation of evolving threats. Following this process avoids biasing the dataset towards short attacks while encoding sustained adversarial presence. Through setting a specific attack-rest cycle for every scenario, a layered dataset is created that allows one to sense both near-term and long-term impact caused by attacks. Furthermore, we included another scenario (Scenario 7), which injects a purely legitimate traffic session (120s). It is mainly crucial in establishing the reference phase of this dataset. This structured resting period acts not only as transitional but also as necessary for analyzing the traffic recovery behavior; posting the sustained cyber-attacks. Such uninterrupted legitimate traffic allows: i) network recovery assessment involves analyzing how MQTT brokers and IoT devices regain stability after a series of attacks, ii) reduction in false positives of detection models allows intrusion detection algorithms to learn and, ultimately, distinguish between real threats and benign network variations, and iii) adversarial adaptation insight involves examining how intertrack intervals influence attack calculations in subsequent stages.

Apart from individual attack time durations, attack scenarios are being tried for an increased duration to model an accumulation of stress on the network by slow build-up instead of immediate unrealistic attack spikes. The very way they are created allows network weaknesses, resource exhaustion points, and performance degradation to manifest on their own without being forced, looking more like authentic IoT environments under continuous cyber-attacks. In fact, by applying real attack scenarios alongside a structured timing mechanism, the methodology ensures that the interaction between adversarial actions and legitimate traffic flows generates a comprehensive, authentic, and strategically designed dataset, ideal for cybersecurity research, intrusion detection model training, and adaptive security studies.

Final validation of the attack execution system is depicted in Fig. 15, which shows the exact timing of the attack, presenting the corresponding scenarios effectively. Beyond this, we can note those preliminary and final attack phases conforming to the time frame anticipated for each, as outlined in Table 6. At the same time, we can see clear bands of legitimate traffic, supporting that this execution framework properly encapsulates attacks and interleaved resting times within its execution. Clearly defined transition points between attack types confirm that the system can distinctly differentiate between the attacks modes. Such accuracy highlights key temporal markers that provide valuable training data for AI classifiers to effectively recognize and categorize cyber threats. This visualization also validates that the data acquisition spends the same time as it carries on an attack. The time periods for all attack segments were captured at precisely the same timestamps and had been validated with label metadata. The goal is to ensure that each time a live attack is presented the system can perform real-time labeling of attack instances, eliminating latency inconsistencies.

### 4.3 *Demonstration of Dataset Reuse Potential: Real-World vs. Simulated MQTT Attack Models*

To demonstrate the usability and reproducibility of the MQTTEEB-D dataset, we present a performance comparison between models trained on simulated versus real-world data, illustrating how dataset characteristics impact machine learning outcomes.

Expanding on the previous discussion of real-time attack execution and structured data collection, this section evaluates the performance of our dataset with MQTTSET, a simulated dataset [18]. Beyond providing detailed dataset explanations, the comparison focuses on how training on simulated data versus real-world data ultimately impacts AI-driven model performance [18].

We first trained the models using MQTTSET, a dataset generated inside a controlled simulated environment. This dataset may include predefined attack patterns but could lack variability, real-world noise, and, most importantly, the execution inconsistencies that arise when live IoT networks are exposed to attacks. For initial training, a traditional AI representation model, the Decision Tree, and a deep learning one, the GRU, were selected. After training on MQTTSET, the models were deployed and tested within our real-time attack execution framework. The aim was to analyze their ability to detect adversarial activities in a dynamic MQTT network. As expected, Models trained solely on simulated data showed degraded performance when exposed to real-time attack scenarios, demonstrating that training on synthetic datasets indeed has its limitations.

**Fig. 16:**
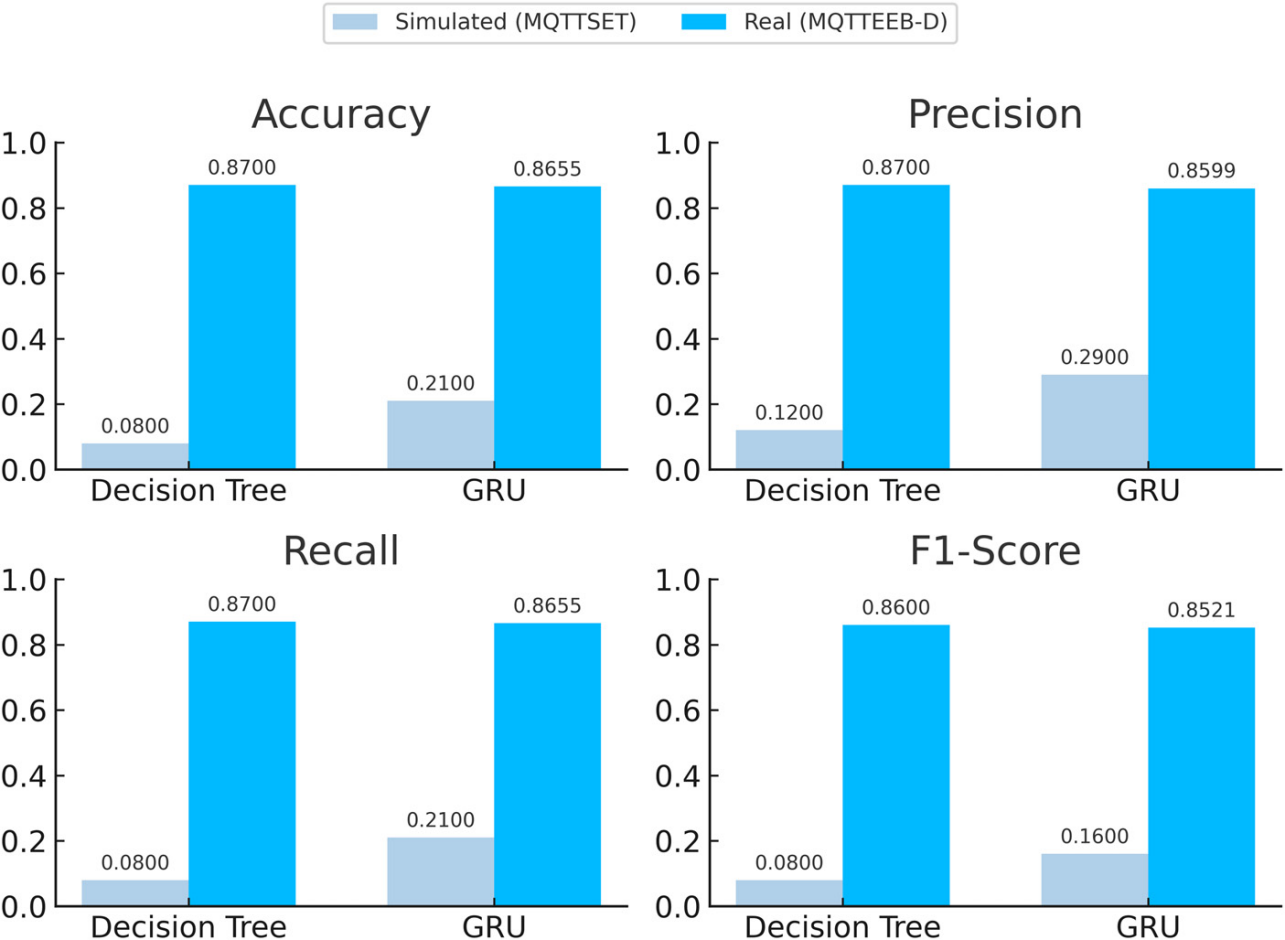
Model Performance Comparison on Simulated vs. Real Datasets.

To address this limitation, the same models were retrained on our MQTEEB-D dataset, which was generated from real MQTT attack executions. This additional training step was necessary because models trained solely using the MQTTSET, lacked exposure to real-world network conditions, leading to degraded performance when faced with actual attack scenarios. By retraining using the MQTEEB-D, the models were able to adapt to network fluctuations, timing inconsistencies, and execution irregularities that were absent in the simulated training phase. Following retraining, the models were re-deployed and tested again in real-time MQTT conditions. Results shown in Fig. 16 demonstrate performance growth across a range of evaluation metrics, including accuracy, precision, recall, and F1-score. Models trained on MQTTEEB-D exhibited significantly better generalization, further emphasizing the role of real-world datasets in cybersecurity research. This highlights the critical role of real-time attack data in developing effective AI-based security solutions for the MQTT-based networks. By working with live attack-derived datasets, instead of simulations, security models will be more reliable in terms of detection and more resistant to adversarial threats. Nesting structured attack executions with synchronized traffic monitoring and dynamic data-set generation led to MQTTEEB-D paving great inroads toward future MQTT security research.

## Limitations

The MQTTEEB-D dataset successfully captured aspects related to security threats in an MQTT-based IoT environment. However, it is limited to MQTT traffic, ignoring security challenges that arise in other IoT protocols, like CoAP or AMQP. With a structured framework, though, it allows room for future expansion to other protocols. Nonetheless, MQTTEEB-D remains an important benchmark for IoT security research, with real-world characteristics that may define its continuity along with wider protocol coverage. Future developments could also incorporate secure cryptographic primitives and implementation-hardening techniques, inspired by recent advances in post-quantum cryptography and side-channel attack resilience, to bolster the security posture of AI-powered intrusion detection systems.

## Future work

• **Expand Protocol Coverage**: Extend the dataset and testbed to include other IoT protocols such as CoAP and AMQP, enabling broader intrusion detection research.
• **Integrate Cryptographic Security**: Implement post-quantum cryptographic schemes and hardware/software secure co-designs to enhance IDS resilience against evolving cyber threats.
• **Real-Time AI Deployment**: Develop adaptive AI-driven intrusion detection models capable of real-time deployment and self-improving through live network feedback.
• **Dataset Expansion**: Future extensions of the dataset are planned to increase the number of instances and capture additional traffic to address scalability considerations. Additionally, the dataset’s modular design facilitates the application of augmentation techniques for researchers who wish to expand or diversify the data further.

## Ethics Statement

The authors confirm that all experiments and data collection were conducted in a controlled laboratory environment, ensuring compliance with ethical research guidelines. The study does not involve human subjects, personal data, animal experiments, or data collected from social media platforms. All IoT attacks executions and traffic monitoring were performed in a secure testbed setup, designed to prevent any adverse effects on operational systems or external networks. Additionally, all security experiments were conducted strictly for research purposes, with no real-world impact beyond the isolated testing environment.

## CRediT Author Statement

**A. Aqachtoul, K. Karam, A. Elamrani, M. Najib, N. Rafalia, M. Bakhouya:** Conceptualization, Methodology, Software Development, Data Curation, Experimentation, Formal Analysis, Investigation, Validation, Results Interpretation, Writing – Original Draft Preparation, Writing – Reviewing and Editing, Supervision, Project Administration, and Funding Acquisition.

## Data Availability

Mendeley DataMQTTEEB-D: A Real-World IoT Cybersecurity Dataset for AI-Powered Threat Detection in MQTT Networks (Original data).
